# Direct Anterior Approach in Crowe Type III‐IV Developmental Dysplasia of the Hip: Surgical Technique and 2 years Follow‐up from Southwest China

**DOI:** 10.1111/os.12713

**Published:** 2020-06-08

**Authors:** Zai‐yang Liu, Jun Zhang, Song‐tao Wu, Zi‐qiang Li, Zhong‐hua Xu, Xia Zhang, Yue Zhou, Yuan Zhang

**Affiliations:** ^1^ Joint Disease & Sport Medicine Center, Department of Orthopaedics, Xinqiao Hospital Army Medical University Chongqing China; ^2^ Department of Orthopaedics People's Hospital of Yunyang Chongqing China; ^3^ Department of Orthopedics People's Hospital of Linshui Guang'an China

**Keywords:** Developmental hip dysplasia, Direct anterior approach, Leg length equalization, Subtrochanteric osteotomy, Total hip arthroplasty

## Abstract

**Objectives:**

To summarize our pioneering surgical practice and clinical outcome of Crowe type III‐IV developmental dysplasia of the hip (DDH) with a direct anterior approach total hip arthroplasty in a single teaching hospital in Southwest China.

**Methods:**

Fourteen patients (15 hips) diagnosed with Crowe type III‐IV developmental dysplasia of the hip were involved in this single‐center retrospective study between 2016 and 2018. A comprehensive surgical procedure, including preoperative planning and algorithms for leg length equalization, intraoperative stepwise soft tissue release, bone defect reconstruction, and an innovative subtrochanteric osteotomy, was described. Furthermore, advancements in intraoperative CT guidance, computer navigation, and nerve monitoring were available for specific demands. The short‐term clinical outcome was evaluated at the endpoint of follow‐up by three patient‐reported functional scales (Harris, WOMAC, and SF‐12 scores), and objective data collected at the clinic, including functional recovery (muscle strength of hip flexor and abductor, correction of the pelvic tilt, leg length discrepancy, and limp), radiographic analysis, and complication occurrence.

**Results:**

The intraoperative variables were carefully recorded. The mean operating times in Crowe type III and IV groups were 115.8 min and 156.2 min, and the median blood loss volumes were 520.5 mL and 810.2 mL, respectively. The general changes in the Harris, SF‐12, and WOMAC scores of the two groups were 46.2, 8.7 and 134.3, respectively, at a mean follow‐up of 25.4 months. Enhanced recovery of hip abductor muscle strength was identified in 85.7% of the population at the third postoperative month. The equalization of leg length and correction of the pelvic tile were observed at the sixth postoperative month, with a 36‐mm decrease in leg length discrepancy. No radiographic evidence of the loosening or migration of the components was observed. A self‐innovated subtrochanteric shortening osteotomy was performed in five patients, and they healed after 6 months. Specific complications included two cases of distal femoral cracks and one case of a periprosthetic fracture needing internal fixation. Two patients received a late iliotibial band release at the 3rd month postoperatively due to progressive genu valgum. No records of infection, dislocation, nerve palsy, bone non‐union, or revision surgery were identified.

**Discussion:**

The direct anterior approach total hip arthroplasty showed potential advantages, including optimum component positioning, improved hip stability, steerable complication rate, and enhanced functional recovery with Crowe type III‐IV DDH. The short‐term outcome is comparable to that of the traditional posterolateral approach.

## Introduction

An increasing number of hip surgeons are now shifting their focus to the direct anterior approach (DAA) in China because it is a naturally minimally invasive approach and a robust method for fast‐tracking surgery^1^. In our hospital, we have treated over 1000 cases of THA using the DAA since 2015, including over 200 cases of developmental dysplasia of the hip (DDH). The advantages of the DAA have been fully exhibited in our practice in terms of rapid recovery, instant weight‐bearing, minimal limitations in daily activities, reduced hospital stay and cost, low dislocation rates, and other benefits^2^.

The DAA is generally recommended for simple and primary hip disorders^3^, such as avascular necrosis, osteoarthritis, femoral neck fracture, and Crowe type I‐II DDH, because these disorders are mild pathologies with soft tissue contractures, bone defects, and articular deformities, where it is convenient to use a regular monobloc prosthesis. However, there is a large portion of patients suffering from severe DDH in the southwest region of China. The morbidity in this region is estimated to be higher than other regions in China according to an informal investigation. Although the exact etiology of DDH is not clear, some intrinsic factors are supposed to contribute to the progress, including genetic (short and thin body shape), cultural (swaddling, kneeling), geographic (mountainous area), climatic (less sunshine, hot and moist), and less development of economy and healthcare.

Although there are well‐established concepts and practices for regular DAA, high‐dislocated DDH, defined as type III‐IV in the Crowe system^4^, is typically considered the true contraindication for DAA because of considerable challenges and controversies caused by factors such as multiple planar deformities^5^, complex lumbar‐pelvis‐hip pathology^6^, special reconstruction and correction techniques^7^, and modular stem implant difficulties^8^. However, there is a high demand for DAA in our patients diagnosed with Crowe type III‐IV DDH, which motivated our exploration of this unknown area, despite very few studies and instructions being available and scant evidence that supports the currently claimed outcome. Therefore, we started our clinical practice with ethical approval in 2015 to answer the following questions:

(i) Can DAA work as a routine approach in treating high‐dislocated DDH?

(ii) What are the key techniques to overcome the challenge in this procedure?

(iii) Can we achieve the aim of enhanced recovery by using DAA to treat high‐dislocated DDH?

The purpose of this retrospective study is: (i) to summarize our pioneering practice of surgical techniques resulting in the treatment of Crowe type III‐IV DDH by the DAA procedure in our teaching hospitals through proposing specific techniques, such as preoperative planning, soft tissue release and exposure, acetabular and femoral preparation, subtrochanteric osteotomy, component placement and reduction techniques, and by introducing intraoperative CT guidance, computer navigation, and nerve monitoring into surgery; (ii) to provide a clear, defined protocol for performing direct anterior approach total hip arthroplasty and to report the pearls and pitfalls in patients with high‐dislocated DDH; (iii) to report the intraoperative variables and clinical outcome at 2 years follow‐up in terms of patient‐reported scales, functional recovery variables, radiographic analysis, and complication occurrence, and further compare the difference between Crowe type III and type IV.

## Methods and Materials

### *Patient Demographics*

We performed a retrospective analysis of all primary DAA procedures completed at our institutions by a senior surgeon (Y.Z.) between 1 July 2016 and 30 June 2018. A total of 209 patients diagnosed with osteoarthritis secondary to DDH were found in the database, and 14 patients (15 hips) diagnosed with Crowe type III‐IV DDH were enrolled. Informed consent was obtained from all patients, and the research ethics committee of our hospital approved our study.

The inclusion criteria were as follows: (i) patients diagnosed with avascular necrosis of the femoral head or osteoarthritis secondary to DDH (Crowe type III‐IV); (ii) patients had progressive hip pain, limping, and lower back pain resulting from DDH, ineffectively treated with conservative measures; (iii) patients underwent THA via direct anterior approach or posterolateral approach in our institution from 1 July 2016 and 30 June 2018; (iv) patients with intact medical record for paralleled comparison between the two surgical treatments; (v) the clinical outcomes could be evaluated by functional improvements, patient‐reported scales, complication, as well as radiographic analysis; and (vi) the design which was consistent with retrospective controlled study. The exclusion criterion were as follows: (i) active infection related to the hip; (ii) severe contracture or stiff hip caused by an infection sequela or previous surgery; (iii) high expectations with pain elimination, gait recovery, and limb length; and (iv) DDH combined with a neurovascular disease such as paralysis caused by cerebral infarction and hemorrhage and limb dysfunction caused by poliomyelitis.

### *Preoperative Planning*

#### *Radiographic Analysis and Templating*

Anteroposterior and lateral hip radiographs are templated prior to surgery for determination of implant size, the level of the femoral neck cut, the anticipated point of femoral offset, and the site of the STO. The acetabular component is placed at the true center (anatomic center), and the femoral neck cut is located approximately 10 mm proximal to the lesser trochanter.

The anteroposterior and lateral lumbar radiographs, as well as the bending X‐ray of the lumbar region, are used to determine the pelvis position in the sagittal plane and the flexibility of the lumbar spine. The standing full‐length X‐ray is used for determining the STO and correcting the LLD.

### *Algorithm of Limb Length Equalization*

Any factor causing LLD and its effect on limb length should be considered, including: hip dislocation; anatomical length of the femur and tibia; condition of the lumbar‐pelvic complex, such as pelvic inclination/flexion/rotation and lumbar scoliosis; intra‐articular deformities, such as coxa vara/valgus and genu vara/valgus; extra‐articular deformities, such as femoral shaft bowing; pathologies of the contralateral hip; and weight‐bearing factors. Although factors might interact in many ways, and the overall evaluation might be complicated^9^, we usually followed a patient‐specific protocol to determine how to balance the LLD.

### *Surgical Process*

#### *Anesthesia and Position*

**Figure 1:**
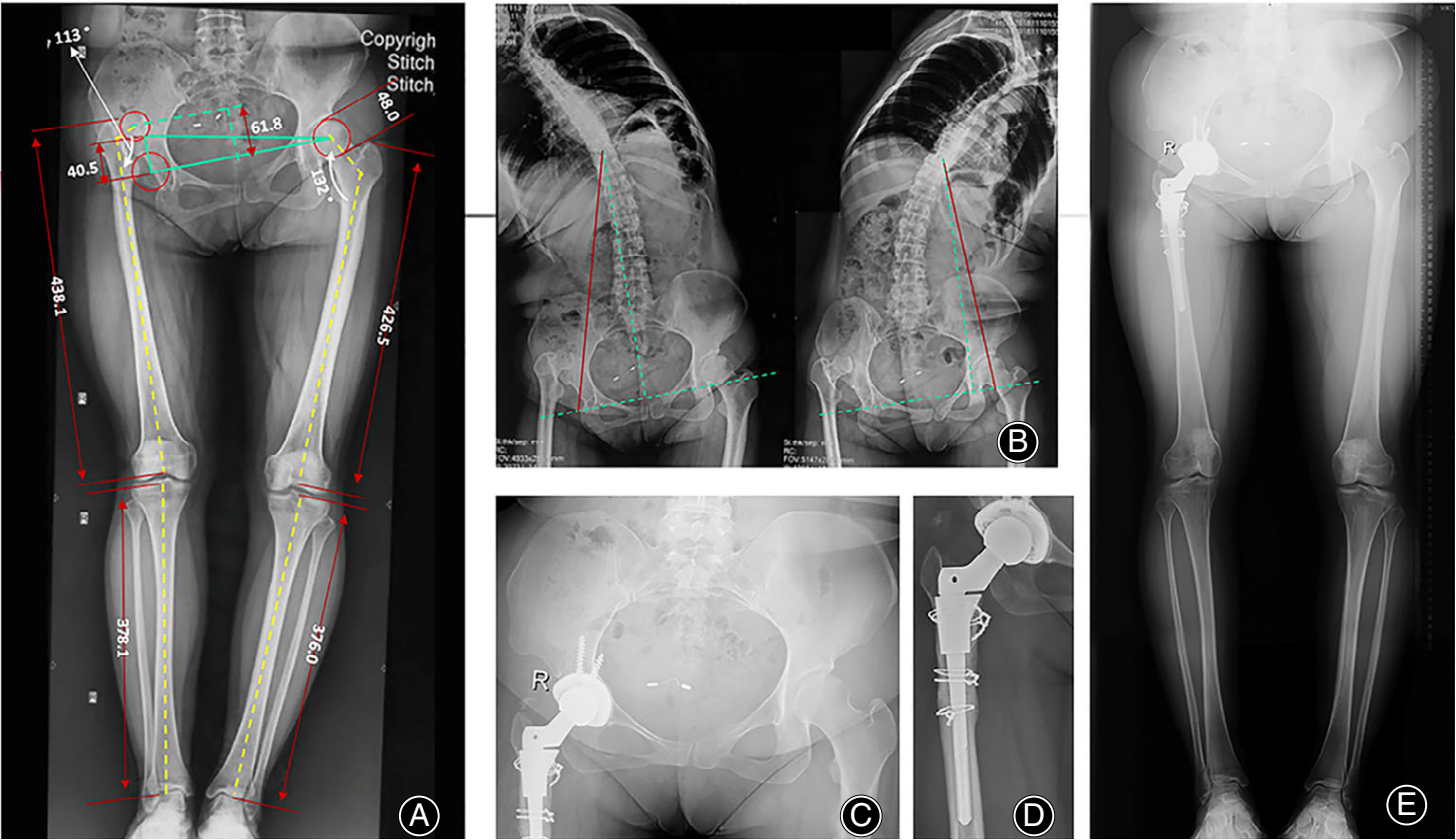
Preoperative planning and postoperative recovery of a Crowe type IV DDH patient treated with DAA THA + STO. (A) A stepwise algorithm for limb length equalization is implemented based on measurements of the hip dislocation height, the anatomical lengths of the lower limbs, the pelvic tilt angle, intra‐articular and extra‐articular deformities, etc. (B) Spinal flexibility determined by bending X‐ray images is important for evaluating the contributions of pelvic tilt and lumbar scoliosis to LLD. (C) The pelvic tilt had been corrected by the 5th month after surgery. (D, E) Bony union of the STO and lower limb length equalization were observed 7 months after surgery.

The patient, under general anesthesia, is positioned in a supine pose on a standard operating table. The pubic symphysis is positioned directly at the flexion point of the table. Both lower extremities are sterilized and draped into the field to allow for direct comparison of limb lengths intraoperatively as well as for stability testing (Fig. 1). Fluoroscopy after anesthesia under axial distraction of the dislocated hip is helpful in estimating the possibility of reduction, the magnitude of soft tissue release, and the need for STO.

### *Approach and Exposure*

**Figure 2:**
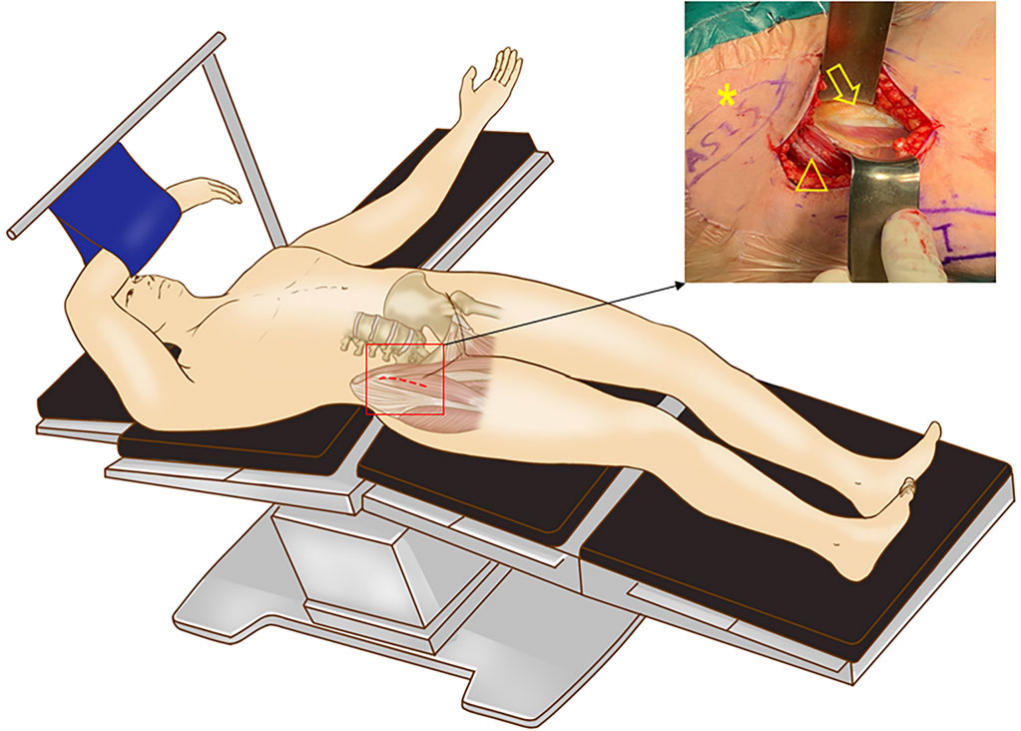
A schematic illustration of the position, approach and exposure in DAA THA. The patient is in a standard supine position with full exposure of the bilateral ASIS and lower limbs. The correct entering approach is in the Hueter interval as shown in the right window (*, ASIS; △, TFL; ↓, rectus femoris).

A standard incision, beginning 2 cm lateral to the ASIS and approximately 8–12 cm in length, is made in the muscle belly of the tensor fasciae latae (TFL). The Hueter interval between the TFL and the rectus femoris can be obtained by routine exposure (Fig. 2). All the ascending branches of the lateral femoral circumflex artery are coagulated to avoid intra‐ and postoperative hematoma. The pericapsular fat, deep anterior capsule, and transtrochanteric process are then identified for an accurate operation. These initial steps are almost the same as the process described by Ong and York *et al*.^3^, ^10^.

The exposure is further developed by a femoral neck cut. The bone is cut 10 mm superior to the lesser trochanter by using the template design to attain a sufficient amount of proximal stability of the sleeve. We preferred to make simple and safe bone cuts in both the vertical and the horizontal planes with a reciprocating saw. The femoral head can be easily extracted from the pseudoacetabulum.

### *Soft Tissue Release*

**Figure 3:**
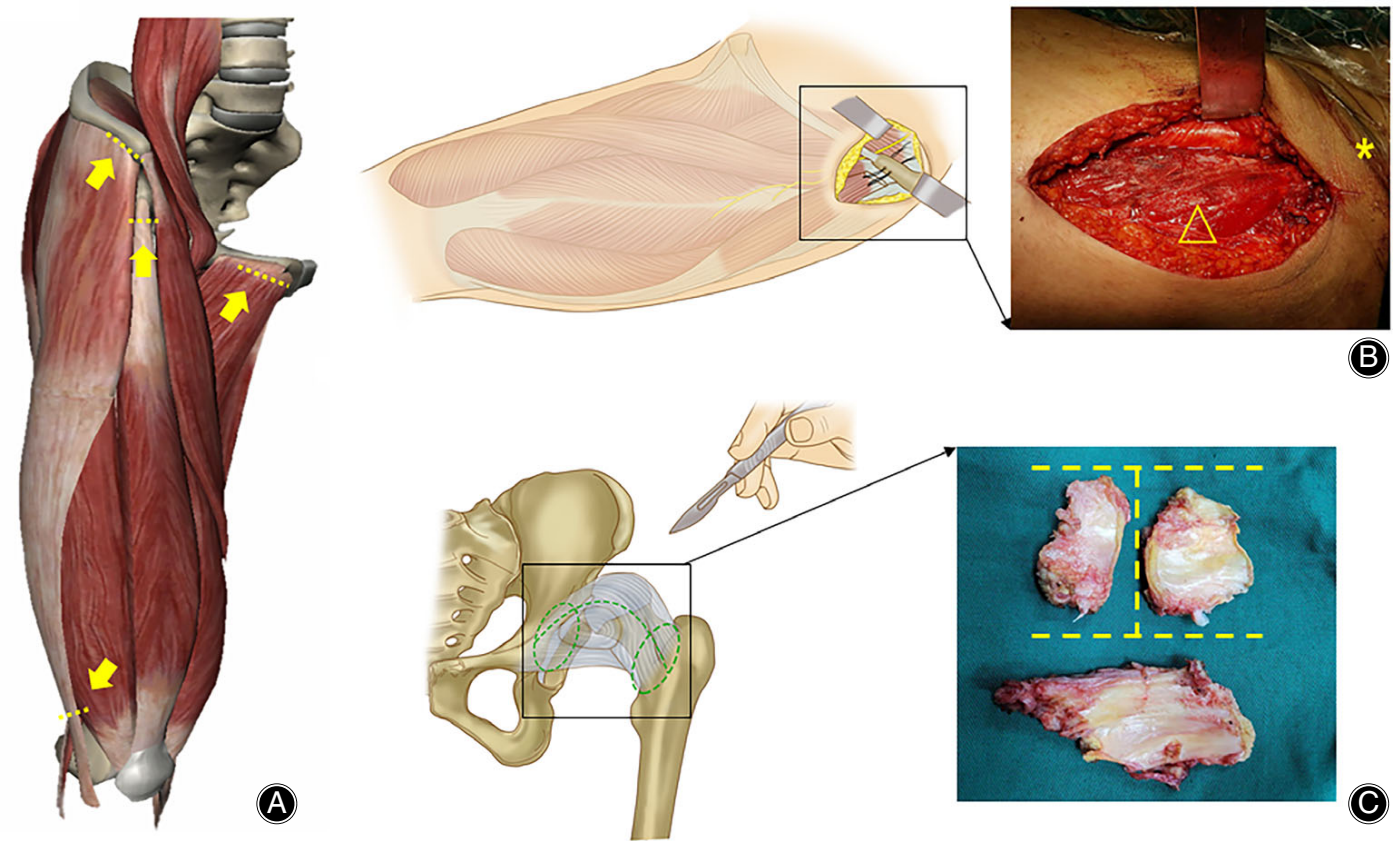
Soft tissue‐releasing techniques of THA *via* DAA in Crowe type III‐IV DDH. (A) The common structures used for soft tissue release usually include tightened adductor tendon, femoral rectus, and distal ITB and attachment of the TFL. (B) A schematic illustration of the peel‐off technique of the TFL and reattachment to the iliac crest by transosseous tunnels with non‐absorbable sutures, which is a simple but effective method to maintain the continuity of the muscle structure and strength (*, ASIS; △, TFL). (C) A schematic illustration of sleeve‐like capsulectomy technique. The green dotted lines indicated the resection region of the capsule. Resected anterior and posterior capsules in a “H”‐manner, and whole posterior capsule can be assembled as a sleeve structure around hip, as shown in the right window.

Since chronic dislocation generally causes soft tissue contractures, adequate release of the soft tissue is crucial to achieve proper joint tension, hip reduction, and limb length equalization. A stepwise procedure including pie‐crusting of the adductor tendon, peeling‐off of the TFL, sleeve‐like capsulectomy, partial release of the psoas tendon and iliotibial band (ITB), removal of the reflex head of the femoral rectus, and subtrochanteric osteotomy (STO) might efficiently retune the soft tissue tension in our practice (Fig. 3A). The tightened fibers of the femoral adductors should be palpated and released with a sterile sharp blade before draping. ITB release 5 cm proximal to the lateral femoral epicondyle can be performed intraoperatively or postoperatively, according to the reduction requirement and physical test results, such as the OBER sign. There is little need for psoas tendon release. For extreme cases such as Crowe type IV or posterolateral dislocation, the proximal exposure can be optionally extended by sharply peeling off the TFL attachment from the iliac crest. The tendon portion of the TFL is labeled with a non‐absorbable suture (Ethicon, DePuy, USA). It is very convenient to reattach the TFL with transosseous tunnels or anchors (Fig. 3B).

A key procedure for soft tissue release is sleeve‐like whole capsulectomy, which is the removal of all portions of the capsule around the femoral head anteriorly, posteriorly, superiorly, and inferiorly. Technically, the anterior capsule can be removed first with a “H”‐shaped resection. Then, the femur is externally rotated to reveal the posterior aspect of the capsule, followed by excisions of the superior, posterior, and inferior capsules (Fig. 3C).

### *Acetabular Reconstruction and Component Placement*

**Figure 4:**
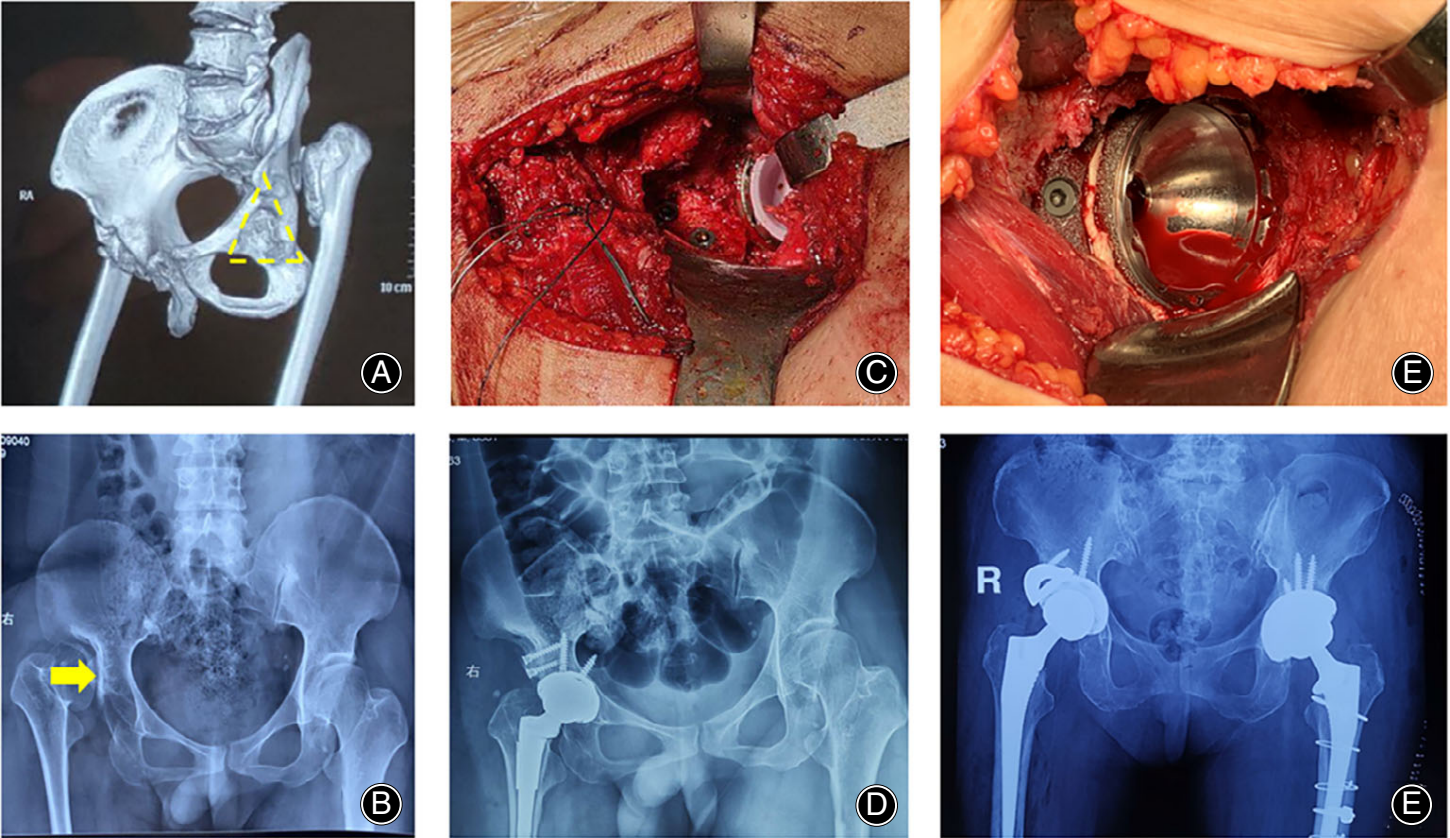
Preparation and reconstruction of the rotation center at the anatomical acetabulum in THA *via* DAA in Crowe type III‐IV DDH. (A) Developmental dysplasia of the acetabulum typically presents as a triangle‐shaped fossa inferior to the pseudoacetabulum. (B) Sclerotic bone between the false and true acetabula usually causes the rotation center to move distally. (C, D) One strategy to address acetabular bone defects is autogenic bone grafting using a healthy part of the resected femoral head when cartilage and sclerotic bone are removed. (E, F) Another strategy is to use tantalum augmentation to enhance the initial stability of the shell (right hip); please note that the left hip received a spontaneous revision due to aseptic loosening.

When the femoral head has been removed, there are three methods for identifying the true acetabulum. The first method is to follow the ligamentum teres distally, which may end at the fossa ovalis. The second method is to identify the inferior capsule, which may be in line with the transverse ligament. The third method is to observe and palpate the bony landmarks, such as the anterior and posterior walls. Typically, there is a triangle‐shaped fossa inferior to the pseudoacetabulum, which contains some fat tissue (Fig. 4A).

Both the anterior and posterior labrum are excised sharply. We preferred to compare the morphology of the true acetabulum with the 3D reconstruction of the CT images or a 3D‐printed model. Here, two typical pitfalls should be addressed in restoring the anatomical rotation center. One strategy is to avoid distalizing the rotation center by removing the sclerotic bone at the superior margin of the true acetabulum (Fig. 4B). Another strategy is to avoid concentric reaming along the true acetabulum, which may result in anteriorization of the rotation center and iatrogenic pelvic discontinuity. We suggest cleaning the osteophytes and sacrificing a small portion of the posterior wall of the true acetabulum using a sharp thin osteotome before reaming. For the Crowe type III cases, the bony coverage is not sufficient for supporting the initial stability of the noncemented shell; thus, the bone defect can be corrected by autografting the femoral head or supporting it with tantalum augmentation (Fig. 4C–F). For bone grafts, removal of the sclerotic bone of the femoral head and cartilage layer of the acetabulum are necessary to achieve bone integration.

**Figure 5:**
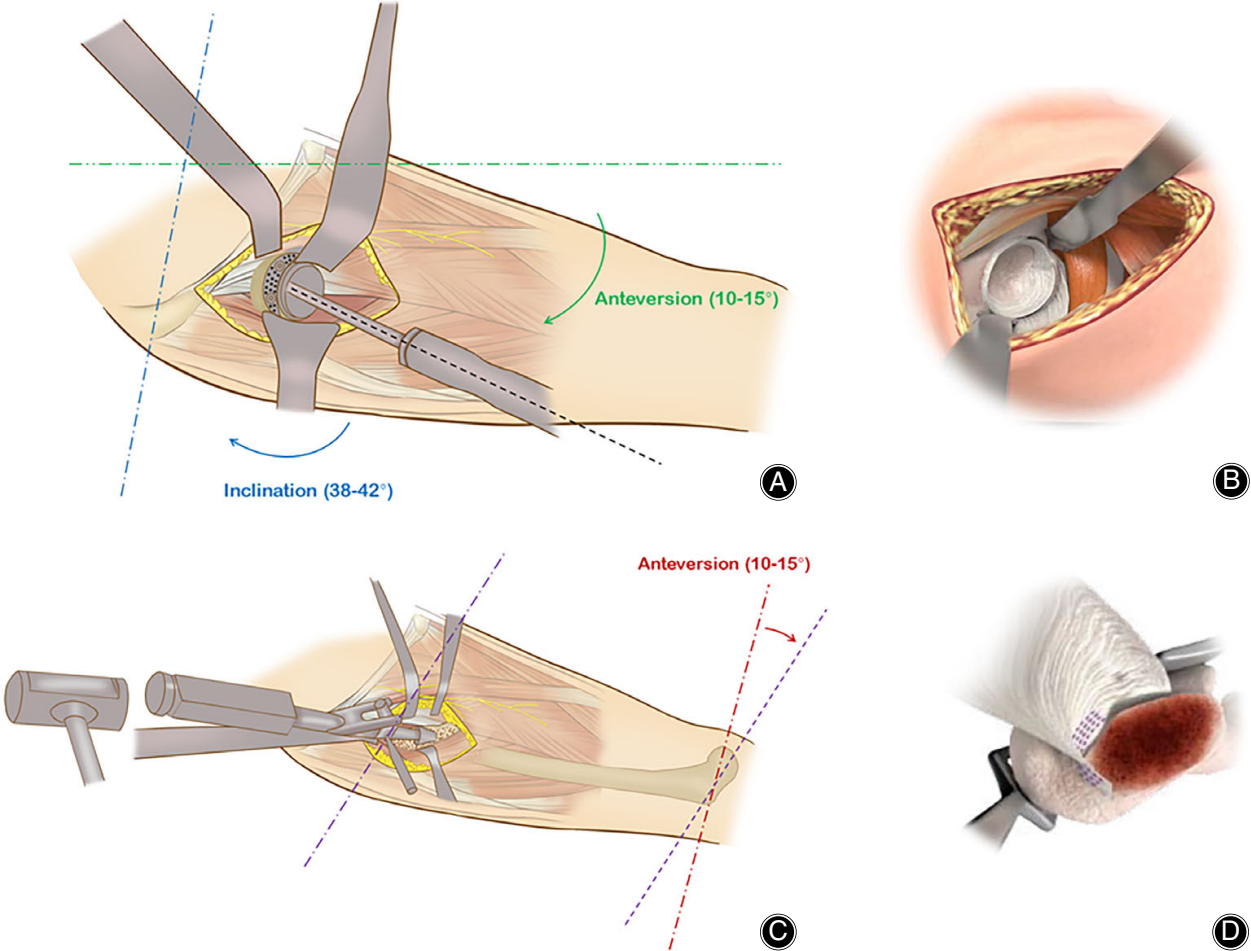
Schematic illustrations of acetabular and femoral component positioning in DAA THA. (A) The acetabular inclination was represented by the intersection angle of shell holder projection (black dotted line) and the connecting line of bilateral ASIS (blue dotted line) in coronal plane, while the anteversion could be determined with the angle between shell holder projection and surgical table plane (green dotted line) in sagittal plane. (B) A fully‐visible exposure of the acetabulum is critical to the accurate position of the shell. (C) The femoral anteversion was determined in reference to the transepicondylar line of the femoral condyle (purple dotted line). (D) The key technique in proximal femoral exposure is the precise and restricted release of the superomedial capsule attachment at the base of the greater trochanter (parallel purple dotted lines).

The reaming and shell implantation techniques are almost the same as those used in traditional anterior approaches^3^, ^10^. We recommend that the cup be placed at 38°–42° of inclination and 10°–15° of anteversion for unilateral DDH. For most bilateral high‐dislocated DDH cases, the inclination and anteversion angles increase with gradual correction of pelvic tilt and abdominal flexion. That is the reason why we chose 30°–35° of inclination and 5°–10° of anteversion for bilateral DDH from the original pelvis. The intraoperative determination of the acetabular position in DAA might be different from that in posterolateral approach. In brief, the inclination could be estimated with the intersection angle of shell holder projection and the connecting line of bilateral anterior superior iliac spine (ASIS) in coronal plane, while the anteversion could be determined with the angle constituted by shell holder projection and surgical table plane in sagittal plane (Fig. 5A, B). Computer navigation is recommended when the pelvis is found in extreme abdominal flexion. On this occasion, the proper position of the acetabular shell should be predicted accurately in preoperative planning and be reproduced by navigation (Stealthstation 7.0, Medtronic, USA) intraoperatively.

### *Femoral Reconstruction and Component Placement*

A typical femoral release and additional implantation of the modular femoral stem can only be started when adequate exposure of the proximal femur is achieved with lateralizing and elevating manipulations when the hip is overextended, adducted, and externally rotated. The femoral procedure begins by releasing the superomedial capsule attachment at the base of the greater trochanter using a reverse “L” manner until the internal side of the great trochanter is visualized (Fig. 5C,D). The incision might extend to the piriformis fossa but not beyond to avoid violating the conjoined tendon. An ideal release is usually indicated by moving the broach straight into the femoral canal without any obstruction from the iliac crest.

**Figure 6:**
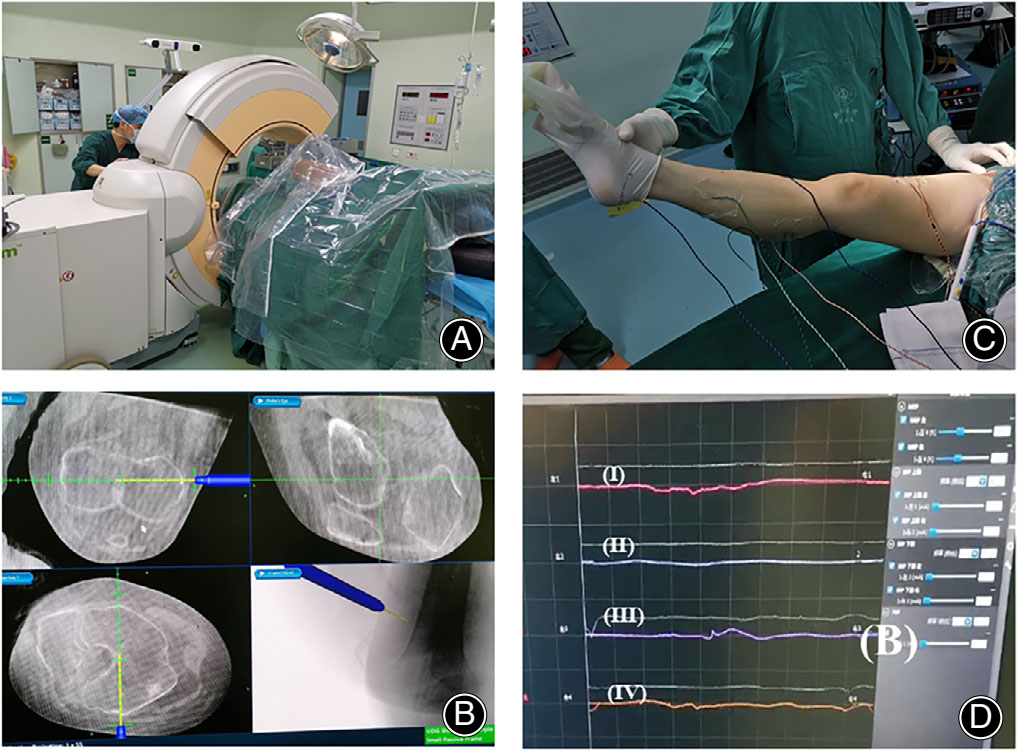
Application of special intraoperative instruments to increase accuracy. (A) Intraoperative CT scanning and navigation are beneficial to precisely determine the degree of femoral anteversion. (B) The transepicondylar line of the femoral condyle is used as a reference, and the femoral anteversion is set to 10º–15°. (C) Introduction of intraoperative nerve monitoring in THA *via* DAA in Crowe type III‐IV DDH. The electrodes are placed into key muscles percutaneously to detect the nerve status during hip reduction. (D) An abnormally evoked potential strongly indicates overlengthening and potential damage to the nerve.

An underlying pitfall here is the need to determine the femoral anteversion by the bony landmarks around the proximal femur. The authors advise using the transepicondylar line of the femoral condyle as the reference and setting the anteversion to 10°–15°. An accurate option is to place a Kirschner wire alone, marked along the anatomical transepicondylar line as a gross reference, with the assistance of intraoperative CT scanning (O‐arm, Medtronic, USA) and a navigation system (S7, Medtronic, USA. Fig. 6 A,B).

A standard proximal broaching and distal reaming process is performed according to the instructions of the modular component^8^, ^11^. Two pitfalls should be mentioned here: one is that the successful implantation of a modular femoral prosthesis such as the S‐rom always requires more elevation and lateral translation of the proximal femur than that of a regular stem. Therefore, the peeling‐off of the TFL attachment or partial release of the piriformis is required for some Crowe type IV cases to minimize the intraoperative risks, such as perforation or fracture of the femur. Another pitfall is the error in reaming the femoral canal. It is crucial to be aware of any deformities below the lesser trochanter, including malalignment and inconsistency of the canal; otherwise, the STO can cause rotational instability of the flute, crack, and even fracture at the distal femur. The implant method of the modular stem is almost the same as the traditional approach.

### *Decision‐Making and Implementation of STO*

The STO is indicated only for necessary functional equalization of the lower limb, the prevention of sciatic nerve injury when the limb is overlengthened (typically more than 5 cm), or alarm from nerve monitoring when reducing the hip.

**Figure 7:**
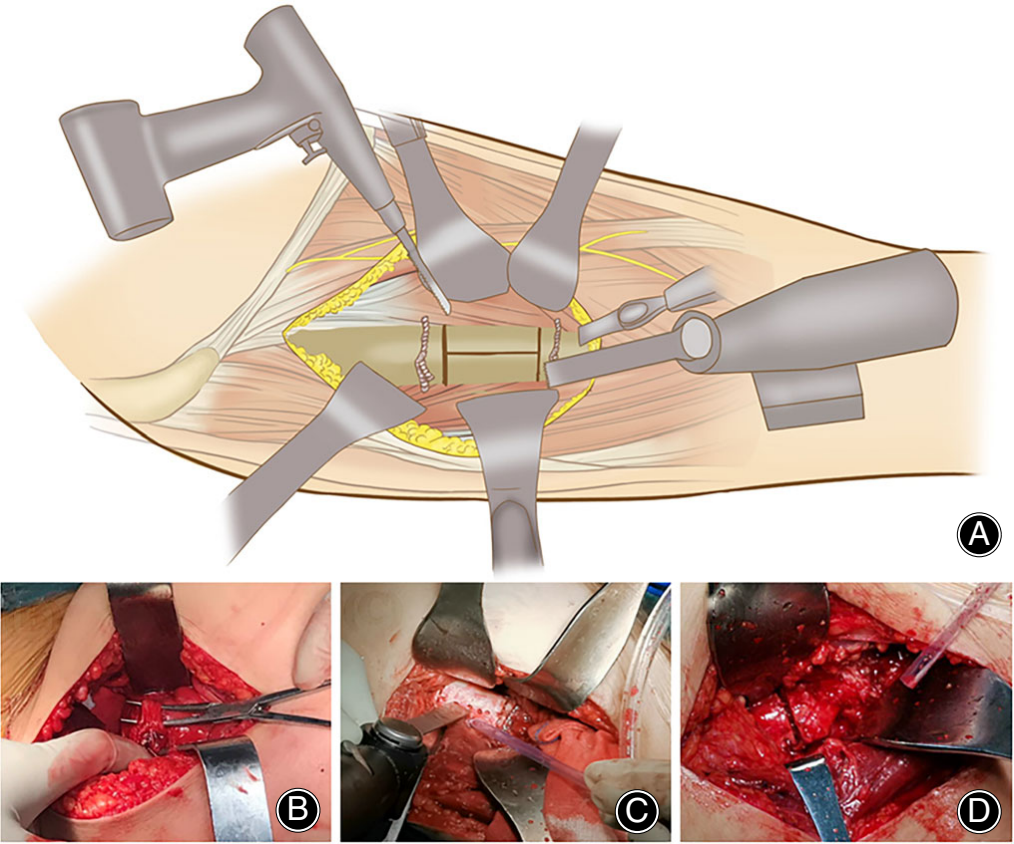
The design and implementation of STO *via* DAA in Crowe type III‐IV DDH. (A) An osteotomy is performed at the anterior cortex of the femur using a suspension saw, at the medial and lateral cortexes using a reciprocal saw, and at the posterior cortex by using a 1.5 mm drill and a thin osteotome. (B) The nerve dominating the vastus lateralis, which crosses the muscular interval between the vastus lateralis and rectus femoris, is identified and protected. (C) When the proximal and distal osteotomies were finished, the vertical open‐up of the osteotomy was achieved by connecting the holes using a narrow suspension saw and sharp osteotome. (D) The final impaction of the femoral stem was completed when close contact ends in the osteotomy and the gap could be filled with autogenous morselized bone.

The incision is usually extended distally approximately 2–3 cm for cases needing an STO. One key is to expose the osteotomy site by splitting the vastus lateralis. However, exposure between the intervals of the vastus lateralis and the rectus femoris may increase the risk for nerve injury (Fig. 7A, B). Excessive caution should be used when identifying the nerve branches dominating the vastus lateralis which cross the above interval. Otherwise, nerve palsy may result in dysfunction of the extension mechanism.

We preferred to perform the osteotomy after implanting the femoral stem since the distal fragment is very difficult to control after the osteotomy. When the starting point for the osteotomy is determined to be 1.0–1.5 cm distal to the lesser trochanter, the proximal and distal segments of the osteotomy are protected by cerclage wire to prevent fracture. Then, the proximal and distal osteotomies are completed with extreme caution. In particular, the anterior cortex of the femur is cut using a suspension saw, the medial and lateral cortexes are cut using a reciprocal saw, and the posterior cortex is penetrated using a 1.5 mm drill and a thin osteotome (Fig. 7C). The bone cut should be made under direct supervision to avoid damaging the titanium flute of the stem. When the osteotomy is performed, the final impaction of the femoral stem is completed when close contact ends in the osteotomy (Fig. 7D). The gap can be filled with autogenous morselized bone derived from acetabular reaming. The cortical strut grafts from the STO are not essential for bone healing.

The function of the STO is not only to shorten but also to correct the anteversion by derotating proximal femur and adjusting the axial alignment by an oblique osteotomy on the proximal femur and a horizontal osteotomy at the distal femur. The latter two methods increase the lever arm of the abductor and further minimize impingement, instability, and limping.

### *Reduction and Closure*

After a 28 mm‐diameter ceramic head is placed, a surgeon‐directed reduction is encouraged. One must be very aware of how to avoid nerve injury, especially for cases without STO. If possible, intraoperative nerve monitoring (NIM‐ECLIPSE system, Medtronic, USA) is very beneficial for detecting nerve status during reduction. An abnormally evoked potential strongly indicates overlengthening of the nerve, and STO or lowering of the femoral rotation center is suggested (Fig. 6C, D).

When the reduction is performed, the patients are always asked to maintain the hip and knee in 50°–60° of flexion to relax the nerve and gradually reach extension within 24 h after surgery. One should be aware of potential pitfalls and test anterior stability in extension or external rotation. Gross estimation of the limb lengths is not needed.

The wound can be closed by stitching the deep fascia of the TFL, the subcutaneous fascia, and the skin with absorbable unknotted sutures (STRATAFIX™ Symmetric, Depuy, USA). Drainage is maintained for 24 h after surgery, and antibiotics are used for the first 24 h.

### *Postoperative Rehabilitation*

The patients are encouraged to walk with crutches for at least 3 weeks. Daily exercise includes hip flexion and abduction and quadriceps muscle strengthening for 12 weeks. For patients undergoing STO, we recommend that they begin walking with toe‐touch weight‐bearing within the first 3 weeks and gradually reach full weight‐bearing 6 weeks after surgery. Oral administration of rivaroxaban is recommended for 5 weeks postoperatively.

### *Outcome Measurement*

#### *Clinical Evaluation*

Clinical follow‐up was performed at 1, 3, 6, and 12 months after the surgery and annually thereafter. Patient‐reported questionnaires and quantitative hip scales were used to assess the pain, function, activity, and health‐related quality of life as following.

#### *Modified Harris Hip Score (mHHS)*

The mHHS is a widely used validated tool to measure the functional capacity of an individual with hip pathology, before and after a surgical procedure. It is a patient‐reported and physician‐completed instrument that consists of subscales for pain severity, function, daily activity, absence of deformity, and range of motion. The score standard had a maximum of 100 points (best possible outcome). A total score <70 is considered a poor score, 70–80 is fair, 80–90 is good, and 90–100 is excellent.

#### *Western Ontario and McMaster Universities Osteoarthritis Index (WOMAC)*

The WOMAC is a commonly used, proprietary set of standardized questionnaires used by health professionals to evaluate the condition of patients with osteoarthritis of the knee and hip. It consists of three subscales: pain (five questions, score range 0–20), stiffness (two questions, score range 0–8), and physical function (17 questions, score range 0–68). The pain was rated as 0 (none), 1 (mild), 2 (moderate), 3 (severe), or 4 (extreme), with the patient completing a separate score for each operatively treated hip. Higher scores represent worse pain, stiffness, and functional limitations.

#### *Short Form 12 Health Survey (SF‐12)*

The SF‐12 is a 12‐item questionnaire used to assess health‐related quality of life from the patient's perspective. It is a reduced version of the SF‐36 in that it has the same number of subscales (physical functioning, role physical, bodily pain, general health, vitality, social functioning, role emotional, and mental health.), but with fewer items per subscale. Scoring (0–100) is derived for each subscale where higher scores represent better health. Norm‐based scoring of each 0–100 scale can then be carried out, which standardizes each subscale to have a mean of 50 and standard deviation of 10. Two aggregate summary measures of the physical component summary (PCS) and the mental component summary (MCS) can be derived. The mean score is set to 50, and score >50 indicate better physical or mental health than the mean and vice versa.

A face‐to‐face inquiry or telephone interview was performed with standardized scripting for all the patients. The walking gait, Trendelenburg sign, and muscle strength of the hip abductor and flexor were recorded by a rehabilitation physician in the clinic. Common and DAA‐specific complications, both intraoperatively and postoperatively, were carefully recorded by one of the authors (Z.L.)

### *Radiographic Analysis*

Anteroposterior radiographs of the pelvis were employed to analyze the position of the components, the deviation of the rotation center, bone‐prosthesis integration, and bone union at the osteotomy site, as well as radiographical signs of loosening, subsidence or migration. In brief, acetabular component loosening was defined as progressive radiolucent lines of >2 mm around the inserted cup, migration, or a change in the position of the cup, which was described by Charnley *et al*.. The alignment and stability of the femoral sleeve and stem were assessed by the method described by Christie *et al*.^12^. Femoral component loosening was evaluated by radiographic analysis as described by Engh *et al*.^13^. The osteointegration of the femoral stem was classified as bone ingrown, fibrous stable, or loose^13^. In addition, the full‐length weight‐bearing X‐view of the lower extremity was used to assess the correction of the pelvic deformity and the equalization of the lower limb.

### *Statistical Analysis*

Two‐sided paired Student's *t*‐test was used to analyze preoperative, intraoperative, and postoperative continuous variables, with *P* < 0.05 considered significant. The data are presented as the mean values with ranges. Statistical analyses were performed using SPSS software version 19.0 (IBM, Armonk, NY, USA).

## Results

### *Demographical Results*

Eight hips were classified as Crowe type III, and seven hips were classified as type IV. The demographics indicated a gender ratio of 12:2 (female/male), a mean age of 34.3 years (range, 18–53), a mean BMI of 22.7 kg/m^2^ (range, 18.5–25.5), a mean symptomatic history of 37.8 months (range, 13–50), and a mean limb length discrepancy (LLD) of 4.1 cm (range, 1.9–6.5). Subgroup data of Crowe type III and Crowe type IV were presented in Table 1, which showed no significant difference in the demographical indicators (*P* > 0.05).

**Table 1:** Comparative and descriptive analysis between subgroups

Indicator	Crowe type III (*n* = 8)	Crowe type IV (*n* = 7)	*P* value
Demographical Index			
Gender ratio (f/m)	5/2	7/0	‐
Age (years)	40.6 (12.7)	28.1 (7.5)	0.34
BMI (kg/m^2^)	25.3 (4.7)	22.4 (5.2)	0.17
Symptomatic history (months)	54.7 (26.9)	27.2 (20.3)	0.09
LLD (mm)	3.5 (0.9)	4.9 (1.2)	0.09
Operational Results			
Surgical time (min)	115.8 (45.0)	156.2 (63.7)	0.02
Blood loss (mL)	520.5 (146.3)	810.2 (157.3)	0.05
STO length (mm)	0	16.5 (4.4)	‐
Bearing surface (COC)	4	7	‐
Clinical Outcome			
Follow‐up period (months)	30.2 (9.8)	23.5 (8.3)	0.03
Changes in HHS	35.9 (18.7)	48.2 (14.1)	0.21
Changes in WOMAC	67.4 (38.3)	54.2 (26.3)	0.04
Changes in SF‐12	7.9 (3.1)	9.2 (3.8)	0.45
LLD (mm)	0.6 (0.4)	0.4 (0.2)	0.27
Complication			
LCFN injury	2	1	‐
TFL injury	3	1	‐
Femoral crack	0	2	‐
Periprosthetic fracture	0	1	‐
Secondary genu valgus	0	2	‐
Radiographical Analysis			
Acetabular inclination (degree)	41.5 (5.2)	36.1 (4.7)	0.10
Acetabular anteversion (degree)	18.1 (3.3)	15.6 (2.5)	0.04
Rotation center deviation (mm) *	5.3 (2.9)	2.1 (2.3)	0.03
Femoral offset deviation (mm) #	3.6 (1.1)	5.2 (3.3)	0.21

* Rotation center deviation was referenced to the anatomical acetabular center.

# Femoral offset deviation was referenced to the contralateral femoral offset.

### *Preoperative Planning*

A patient‐specific protocol was established to achieve limb length equalization. Take the case in Figure 1 as an example, a stepwise algorithm to balance limb length was made based on measurements on the bilateral hips in the following steps. Step 1: Determine the dislocation height. Reduction increased the limb length by 61.8 mm in this case. Step 2: Measure the anatomical length of the lower limb. Most high‐dislocated hips exhibited overgrowth of the femur in ipsilateral side (11.6 mm in this case). Step 3: Determine the magnitude of the pelvic tilt and lumbar scoliosis. Most cases of Crowe type III‐IV DDH exhibited pelvic inclination ipsilaterally. Correction of the inclination reduced the limb length by 40.5 mm in this case, by a simple trigonometric calculation. However, for patients with stiff or degenerated lumbar spines, this compensation might be ignored. Compensations can be easily identified in bending X‐rays of the lower spine and pelvis. Step 4: Adjust the length caused by intra‐articular and extra‐articular abnormalities. For example, a standard femoral stem (135° in CCD) might mildly increase limb length in cases of coxa vara. The peri‐articular abnormality was neglected in this case, because of a normal CCD angle in the contralateral hip.

### *Surgical Process*

A POROCOAT porous, noncemented acetabular shell (PINNACLE) (DePuy, Indiana, USA) was used in 13 hips, and a GRIPTION PINNACLE was used in two hips. All of the acetabular shells were 44 mm in diameter and coupled with a ceramic‐on‐ceramic liner and a 28‐mm head. A fluted modular femoral stem (S‐rom, DePuy, Indiana, USA) was used in 14 hips, while a single‐wedge tapered stem (TRI‐LOCK) (DePuy, Indiana, USA) was used on one hip. For femoral modular components, triangle sleeves were used for 11 patients, and cone sleeves were used for four patients. The median diameter of the distal part of the S‐rom stem was 9 mm (range, 7 to 12). The frequency of ceramic‐on‐ceramic (COC) bearings in Crowe type III and Crowe type IV was 4 vs 7. The average operating times in the two groups were 115.8 min (range, 72‐136) and 156.2 min (range, 98‐173) from incision to closure (*P* < 0.05, Table 1). The intraoperative blood loss was 520.5 mL (range, 325‐650) and 810.2 ml (range, 580‐1000) for Crowe type III and IV, respectively (*P* < 0.05, Table 1). The overall transfusion rate was 60.0%. Five patients with Crowe type IV needed a shortening STO, which had a median osteotomy length of 16.5 mm (range, 10‐27).

### *Clinical Outcome*

#### *Results from mHHS, WOMAC, and SF‐12*

The average follow‐up period was 25.4 months (30.2 months in Crowe type III and 23.5 months in Crowe type IV, *P* < 0.05). The mean increments of the Harris and SF‐12 scores demonstrated no statistical difference between subgroups of Crowe type III and Crowe type IV (*P* > 0.05, Table 1). However, the average value in the WOMAC score exhibited greater decrease in Crowe type IV as compared to Crowe type III (*P* < 0.05, Table 1), which suggested the patients had better recovery in pain, stiffness, and hip function.

#### *Muscle Strength, Correction of LLD, Limp, and Genu Valgum*

The percentage of patients whose hip abductor muscle strength reached scale V was 85.7% three months after surgery and 100% at the last follow‐up. The pelvic tilt was corrected at 6.2 months (range, 3.4‐10.8), and the mean LLD in the 14 patients was reduced from 4.1 cm (range, 1.9‐6.5) to 0.5 cm (range, 0 to 1.3) by subjective judgment from the patients (Fig. 1 C‐E). There was no significant difference in the subgroup comparison in terms of the recovery of muscle strength, pelvic tilt, and LLD at the final follow‐up. Thirteen patients had a positive Trendelenburg sign preoperatively, and only one had a slight limp at the last follow‐up. Two patients with Crowe type IV were treated with secondary iliotibial band release at the clinic because of the development of genu valgum. The tightened fiber at the supracondylar level was lengthened in a pie‐crusting manner under topical anesthesia, and the patients felt relaxed instantly. The valgus deformity disappeared over the following 2 months.

### *Complication and Treatment*

**Figure 8:**
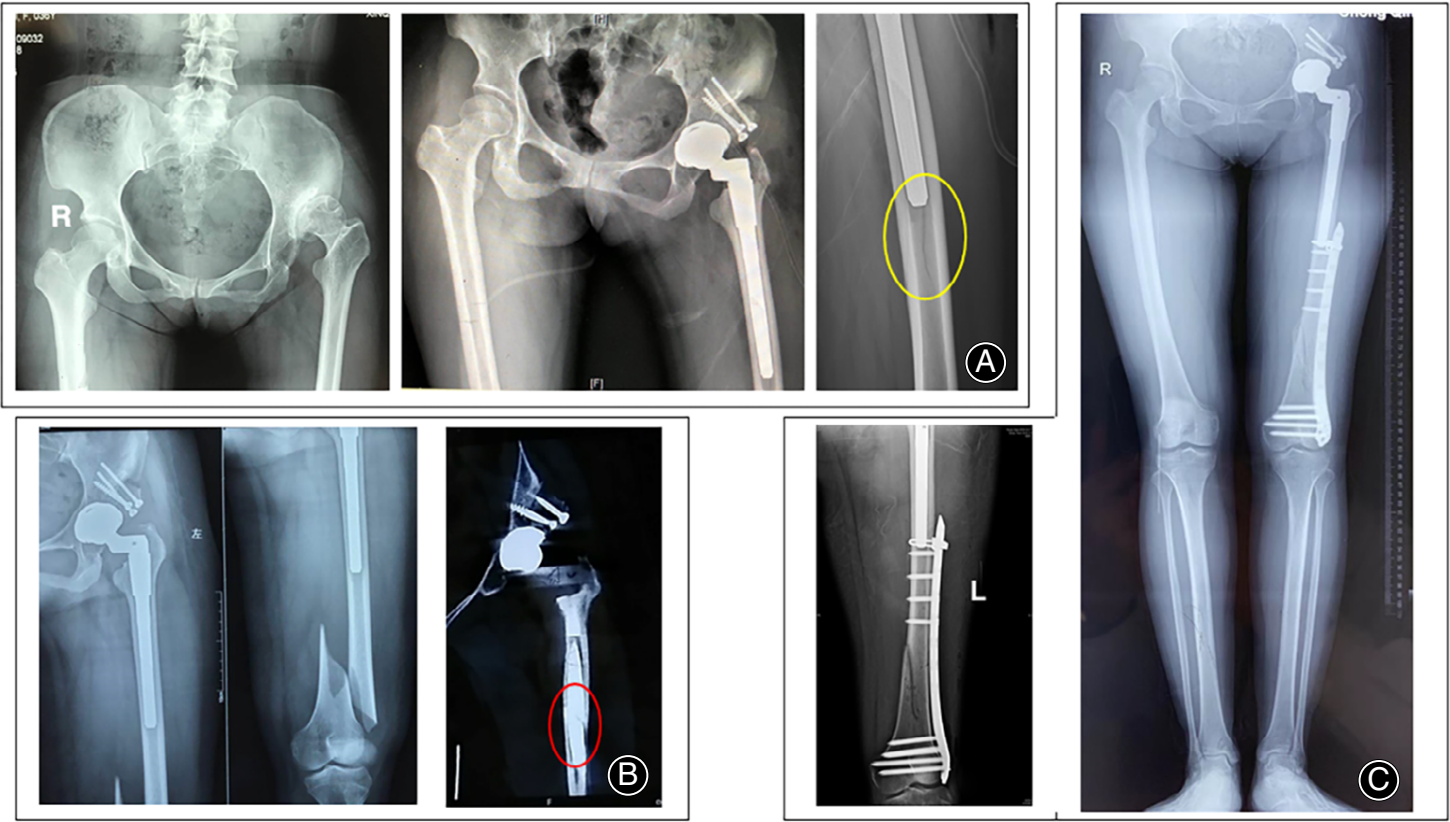
One specific bony complication related to THA *via* DAA in Crowe type III‐IV DDH. (A) Preoperative and postoperative pelvic AP X‐ray of a 37‐year‐old female patient diagnosed with Crowe type IV DDH. A bony crack was found in the distal femur at the distal tip of the stem (yellow circle), and the patient was asked to limit weight‐bearing for 6 weeks. (B) A fall when the patient descended some stairs resulted in a periprosthetic fracture (Vancouver type C) and a half‐slot breakage at the distal (red circle, stem size of 11 mm in diameter). (C) The patient was treated by MIS‐ORIF with a condylar locking plate, screws and cables. The fracture had healed 5 months after surgery without any sequelae to the joint function.

The common complications in this series included four cases of TFL injury, defined by interruption of one third of the muscle belly (no special treatment required due to asymptomatic performance), three cases of LCFN injury (complete recovery after 1‐2 months with Vitamin B administration), and two cases of overlengthening of the leg (full recovery after 6‐8 months with progressive weight‐bearing and active rehabilitation). The specific complications related to high‐dislocated DDH included two cases of distal femoral cracks, the patients were asked to prolong the use crutches and to take half weight‐bearing training within the first months after surgery. However, one of the cracks developed into a periprosthetic fracture (Vancouver type C) and a half slot breakage at the distal stem. The patient was treated by minimally invasive surgery of open reduction and internal fixation, using a condylar locking plate, screws, and cables (Fig. 8A–C). One patient with Crowe type IV was converted to a posterolateral approach due to reduction failure. There were no records of infection, dislocation, nerve palsy, DVT, hematoma, osteotomy non‐union, heterotopic ossification, or component loosening and revision.

### *Radiographic Analysis*

Postoperative anteroposterior radiographs of the pelvis showed encouraging results on the accuracy of acetabular inclination (41.5° in Crowe type III and 36.1° in Crowe type IV, *P* > 0.05) and anteversion (18.1° in Crowe type III and 15.6° in Crowe type IV, *P* < 0.05). A total of 86.6% of the acetabular component was located in functional safe zone^14^. The rotation center deviation was 2.6 mm (range, −2.5 to +6.5) in reference to the anatomical center, and the subgroups comparison showed less deviation in Crowe type IV (*P* < 0.05). In contrast, the femoral offset in Crowe type IV showed greater deviation as compared with that in Crowe type III, but the difference was considered as non‐significant (*P* > 0.05, Table 1). There was no loosening of the acetabular shell and femoral stem at the final follow‐up, as evidenced by the absence of radiolucent lines, spot welding, osteolysis, and stress shielding signs. Neither significant subsidence, defined as a vertical distance of 3 mm from the shoulder of the stem to the tip of the greater trochanter, nor malalignment, defined as varus or valgus of the sleeve and stem, were found according to the method described by Christie *et al*.. All proximal sleeves were classified as bone ingrown by fixation using the Engh classification system. All bone unions at the STO site were observed 6 months after surgery (Fig. 1D).

## Discussion

This study aims to present a well‐defined and standardized protocol for performing direct anterior approach total hip arthroplasty in treating high‐dislocated DDH. We think it should be established on systemic understanding of the different philosophies of the current surgical approaches. Despite satisfactory middle‐ to long‐term clinical results obtained for Crowe type III‐IV DDH by a posterolateral approach^10^, ^15^, ^16^, some deficiencies have been discussed in many publications. First, invasion of the short externals, although properly repaired, might increase the risk of instability and dislocation^17^. Second, interruption of the branch of the femoral artery impairs osteointegration on the host bone‐prosthesis interface and increases the risk of nonunion at the osteotomy site^18^. Given these points, the DAA seems to be advantageous for high‐dislocated DDH.

Another vital issue differentiating DAA and posterolateral approach is the status of lumbar‐pelvic‐hip complex, which was considered to play a pivotal role in decision‐making for Crowe type III‐IV DDH^19^. Specifically, sagittal imbalance of the pelvis usually causes anterior or posterior impingement during sit‐to‐stand transitions^6^. Therefore, a functional pelvic position can be more effectively achieved in the supine position with a DAA than in the lateral decubitus position with a posterolateral approach. A functional pelvic position is considered crucial for reproducible and safe acetabular component sizing and positioning. Therefore, the superiority of the optimum component position, impingement‐free motion, and stability of the hip can be easily obtained with a DAA^20^, ^21^. To our knowledge, there are very few studies reporting THA via the direct anterior approach in treating Crowe type III‐IV DDH. Thus, this study, reporting technical instructions and clinical outcomes, is imperative for overcoming the current difficulties. However, a remarkable study conducted by Oinuma *et al*. reported fair outcomes in 12 hips of nine patients diagnosed with Crowe type IV dysplasia in Japan from 2006 to 2011^22^. In this study, the patients were treated by DAA with STO. The mean operative time was 167 min with a mean blood loss of 1171 mL. Three of the cups deviated “Lewinnek” safe zone, and one dislocation occurred at the early stage after surgery. The functional improvement in the hip and seemed to be acceptable at a mean follow‐up period of 3.7 years. No Trendelenburg test or a Duchenne limp was observed. No radiolucent line nor loosening of the components, and non‐union of the osteotomy site were found by radiographic evaluation. The limitation of this study is the lack of detailed information regarding surgical decision‐making and technique pearls and pitfalls, as well as undesirable intraoperative record.

Here, we have illustrated an efficient and reproducible way to ensure favorable surgical performance of the DAA in high‐dislocated DDH patients. Although there is a definitive learning curve to start treating DDH via DAA, we think the success still relies on careful preoperative planning and intraoperative implementation. It is a good advice for most surgeons to initiate the attempt with Crowe type I‐II DDH cases, then progress to Crowe type III, even type IV. We also believe that thorough technical instructions, specifically the pearls and pitfalls, and the complication prevention strategies presented in our study, are significant advancements. Furthermore, the introduction of intraoperative CT guidance, computer navigation, and nerve monitoring significantly improved the precision of the surgery.

Another aim of this study is to observe the clinical outcome of high‐dislocated DDH patients by our techniques and procedure in treating high‐dislocated DDH by DAA. The operative variables in our series seem to be better in terms of shorter operative time and hospital stay, less blood loss, and a lower complication rate than those in the report by Oinuma^22^. We think the results are related to the soft tissue‐sparing nature of this approach and the fact that our previous work and surgical technique were formulated by treating over 200 cases of DDH in Crowe types I and II. Besides, the patient‐reported results from mHHS, WOMAC, and SF‐12 scores indicated that satisfactory functional recovery could be obtained at an early period of follow‐up. This can be further evidenced by effective correction of the pelvic tilt, LLD, and limping, as well as osteointegration at the bone‐prosthesis interface by radiographic analysis. Furthermore, the complication rate in this consecutive series is quite acceptable. The common complication in this series is identical to that of other primary THA methods completed with the DAA. Similarly, the specific complications related to our series in Crowe III‐IV DDH cases treated by the DAA were not substantially different from those of Crowe III‐IV DDH cases treated by the traditional posterolateral approach.

An interesting finding in this study was that some measuring differences were identified in radiographic analysis. Although postoperative radiographs showed satisfactory results on the accuracy of position of the components, we still found greater anteversion of the shell and increased proximal deviation of the rotation center in Crowe type III. This could be partially explained by anatomic variation in acetabular morphology in Crowe type III DDH, such as the bone defect at the superior rim of the acetabulum, or the pseudoacetabulum formation, which usually causes intraoperative error in acetabular preparation. Thus, computer‐navigation or patient‐specific instrumentation might reduce the incidence of the error. Similarly, another prominent error in femoral reconstruction is the insufficient recovery of the femoral offset in Crowe type IV, which was referenced to the contralateral femoral offset. The most common cause of this deviation is the developmental deformity at the proximal femur, such as over‐anteversion or ante‐torsion of the femoral neck, or overhang of the greater trochanter. In this scenario, the oblique or de‐rotational combined shortening osteotomy, as well as a high‐offset modular stem, should be considered to achieve the aim of anatomical reconstruction.

In conclusion, this study has proven that the DAA is a feasible and even superior option for treating Crowe type III‐IV DDH compared to the posterolateral approach in terms of minimal invasiveness and fast recovery, less interruption of the GM and short rotators, improved stability of the hip, minimal complication rates, and optimal positioning of the components.

The limitation of this study is a deficiency in the strength of the evidence due to the small sample size and the uncontrolled and retrospective study design that included patients from a single hospital in Southwest China. Mid‐ and long‐term clinical data are unavailable at present. This limitation needs to be addressed by conducting a multicenter, prospective cohort study with a large sample population in our ongoing work.

## Ethical Review Committee Statement

This research was approved by the ethics committee of the Second Affiliated Hospital of the Army Medical University (grant no. 2015–099‐01).

## Contributions to the Work

Study design and surgery performed by Yuan Zhang, Yue Zhou. Data collection and analysis by Zaiyang Liu, Ziqing Li, Songtao Li, Zhonghua Xu, Jun Zhang, Xia Zhang. Manuscript preparation by Zaiyang Liu, Zhonghua Xu, Jun Zhang.
